# Data on MRI brain lesion segmentation using K-means and Gaussian Mixture Model-Expectation Maximization

**DOI:** 10.1016/j.dib.2019.104628

**Published:** 2019-10-10

**Authors:** Ju Qiao, Xuezhu Cai, Qian Xiao, Zhengxi Chen, Praveen Kulkarni, Craig Ferris, Sagar Kamarthi, Srinivas Sridhar

**Affiliations:** a Department of Mechanical and Industrial, Northeastern University, Boston, MA, USA; b Department of Bioengineering, Northeastern University, Boston, MA, USA; c Department of Pharmacology, Yale University, New Haven, CT, USA; d Department of Orthodontics, Shanghai Jiaotong University, Shanghai Ninth People's Hospital, Shanghai, China; e Department of Psychology, Northeastern University, Boston, MA, USA; f Department of Physics, Northeastern University, Boston, MA, USA

**Keywords:** Ischemic stroke, Lesion, Magnetic resonance image (MRI), Segmentation

## Abstract

The data in this article provide details about MRI lesion segmentation using K-means and Gaussian Mixture Model-Expectation Maximization (GMM-EM) algorithms. Both K-means and GMM-EM algorithms can segment lesion area from the rest of brain MRI automatically. The performance metrics (accuracy, sensitivity, specificity, false positive rate, misclassification rate) were estimated for the algorithms and there was no significant difference between K-means and GMM-EM. In addition, lesion size does not affect the accuracy and sensitivity for either method.

**Table:** Specifications Table

Subject area	Biology
More specific subject area	Magnetic Resonance Imaging Segmentation
Type of data	image, graph, figure
How data was acquired	Raw data were from ischemic stroke lesion segmentation online database. Segmentation data were acquired using K-means and Gaussian Mixture Model-Expectation Maximization algorithms.
Data format	analyzed data
Experimental factors	All images were normalized and co-registered for all subjects
Experimental features	The segmentation labels were determined using K-means and GMM-EM
Data source location	Raw data at: http://www.isles-challenge.org/ISLES2015/; owned by ISLES. Lubeck, Germany. Segmentation data: Northeastern University, Boston, MA, US; segmentation data is included in this article and can be downloaded from this article
Data accessibility	Segmentation data is included with this article

**Table:** 

**Value of the Data** • These data provide automatic segmentation of lesion in MRI using K-means and GMM-EM. • These data evaluate the performance of K-means and GMM-EM algorithms regarding MRI segmentation. • The data show that lesion size does not affect the performance of K-means and GMM-EM in lesion segmentation

## 1 Data

Magnetic Resonance Imaging (MRI) data were pre-processed. Instead of using the conventional method to manually segment lesion [1] which is time-consuming, inaccurate, and subjective, K-means and Gaussian Mixture Model-Expectation Maximization (GMM-EM) algorithms were applied to automatically segment lesion regions from the rest of brain tissue in MRI. The data included here provides the lesion segmentation results using K-means (dataset as K-means estimated labels.mat) and GMM-EM (GMM-EM estimated labels.mat) as well as the ground truth mask (ground truth mask data.mat). These three datasets are the estimated labels and ground truth mask of brain regions for all 28 subjects.

**Fig. 1:**
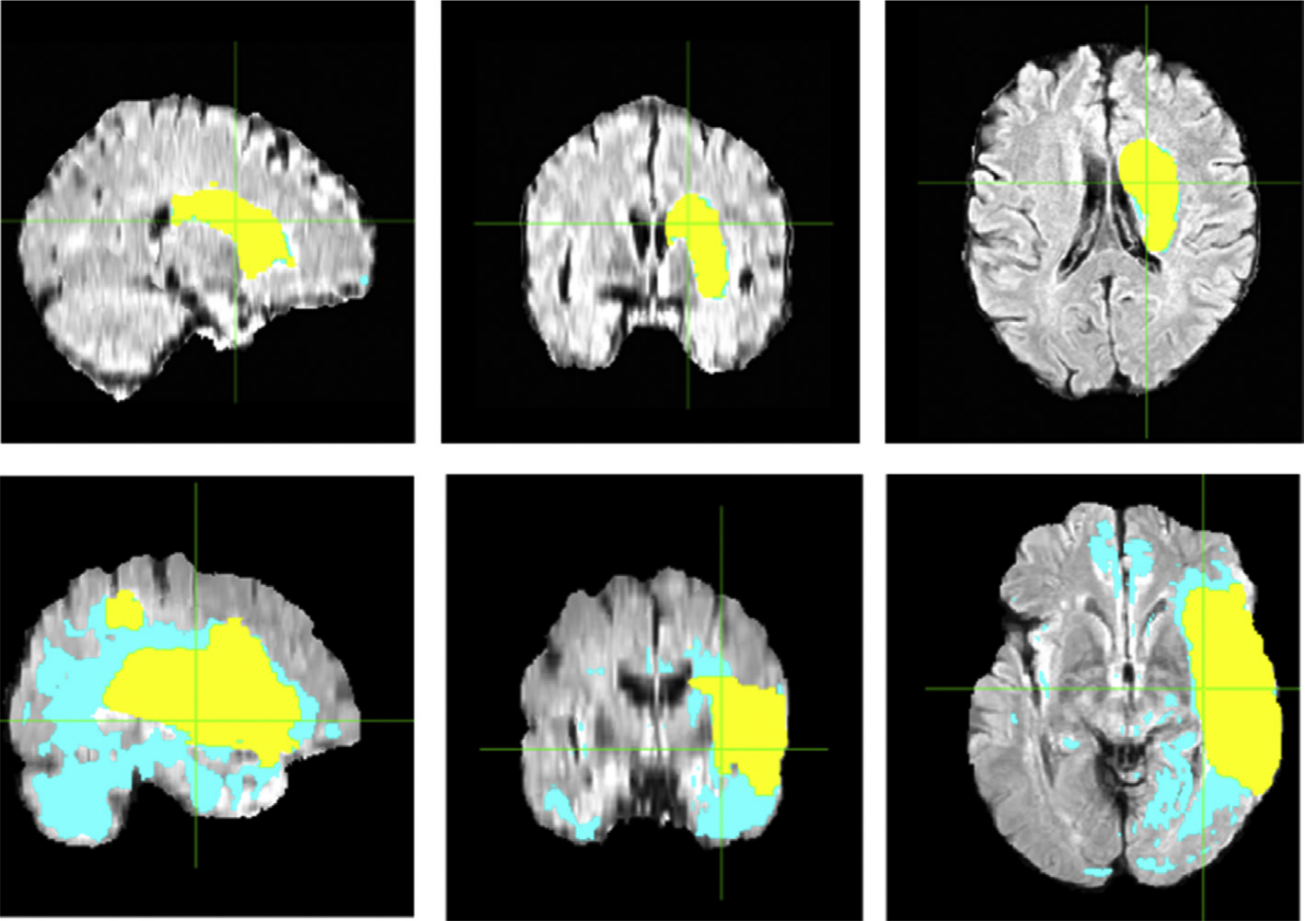
Brain lesion segmentation using K-means. A representative brain lesion segmented using K-means with accuracy of 99.27% (top row). Yellow colored mask is ground truth, while is overlaid on top of the estimated label in blue. A representative lesion segmentation using K-means with accuracy of 56.96% (bottom row).

Fig. 1 shows the brain lesion segmentation using K-means. The best performance (Fig. 1 top row) shows that the estimated lesion regions (light blue) and the ground truth (yellow) match very well with the accuracy of 99.27%. The accuracy of K-means varies from subject to subject. And for some subject, the accuracy is only 56.96% (Fig. 1 bottom row).

**Fig. 2:**
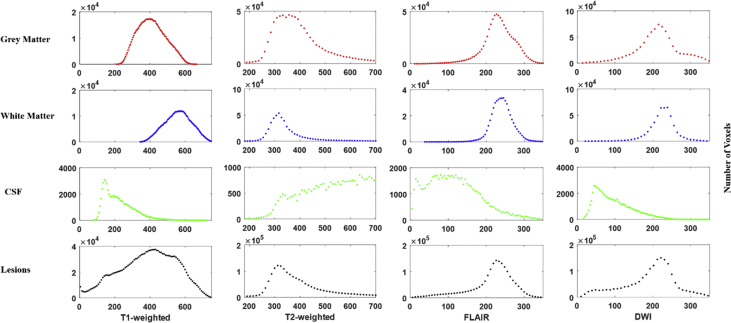
Signal intensity histogram of GM, WM, CSF, and lesion (if present) in T1-weighted, T2-weighted, FLAIR, and DWI MRI of a representative subject.

**Fig. 3:**
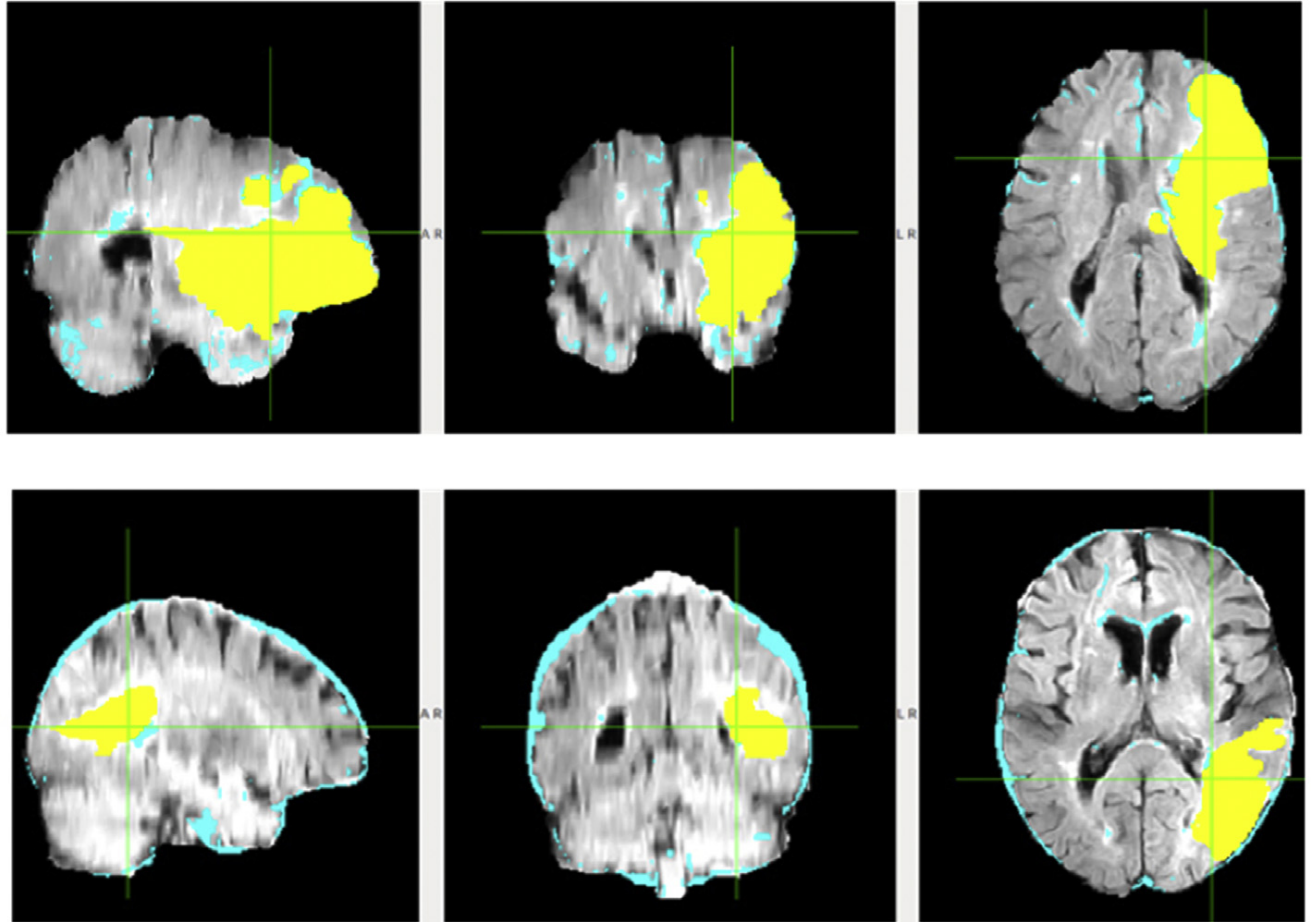
GMM-EM brain segmentation visualization. The best performance (top row) of GMM-EM have the accuracy of 95% and representative subject with accuracy of 89.02% (bottom row) shows GMM-EM misclassifies edge of the brain as lesion.

GMM-EM is applied to segment brain lesion, since in each MRI image modality, the intensity of four different brain tissues follows Gaussian distribution approximately as shown in Fig. 2. The segmentation shows GMM-EM works well with the average accuracy of 85%. The estimated lesion regions (light blue) matches the ground truth lesion regions (yellow) well for the best performance subject (Fig. 3 top row) with the accuracy of 95%. While for some subjects, GMM-EM does not segment lesion correctly with healthy regions misclassified as lesion regions. Fig. 3 bottom row shows representative subject with accuracy of 89.02% and the edge of the brain is misclassified as lesion.

**Fig. 4:**
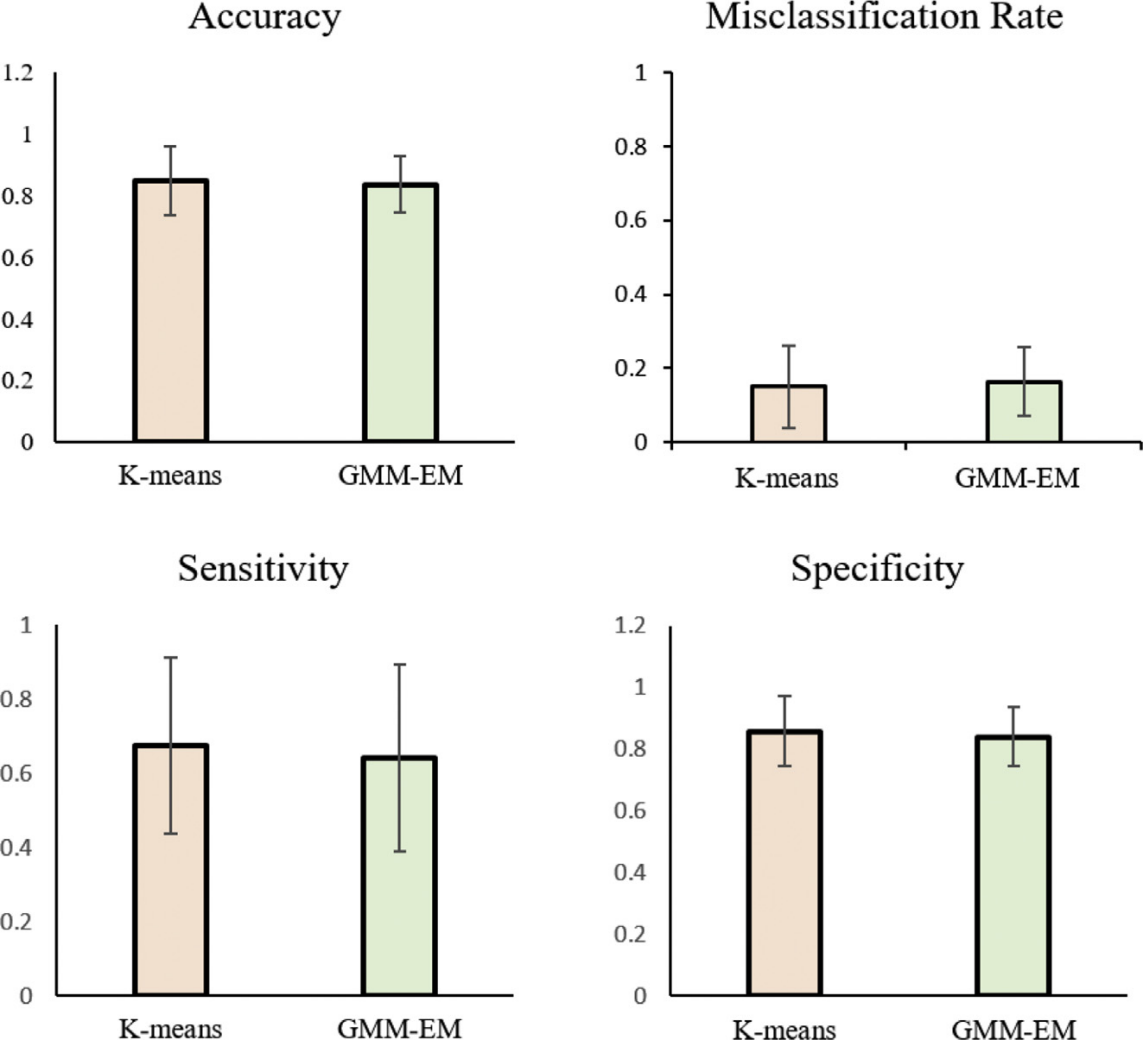
Algorithm performance evaluation and comparison. There is no significant difference between K-means and GMM-EM in accuracy, sensitivity, and specificity.

The performance metrics (accuracy, misclassification rate, sensitivity, specificity, and false positive rate) were calculated for both K-means and GMM as shown in Fig. 4. The accuracy, sensitivity, and specificity of K-means are 85 ± 11%, 67 ± 24%, and 86 ± 11% specifically. The accuracy, sensitivity, and specificity of GMM-EM are 84 ± 9%, 64 ± 25%, and 84 ± 10% specifically. There is no significant difference between K-means performance and GMM-EM performance (p-values of accuracy, sensitivity and specificity are: 0.6645, 0.7647, 0.5479). In addition, both K-means and GMM-EM performance varies from subject to subject.

**Fig. 5:**
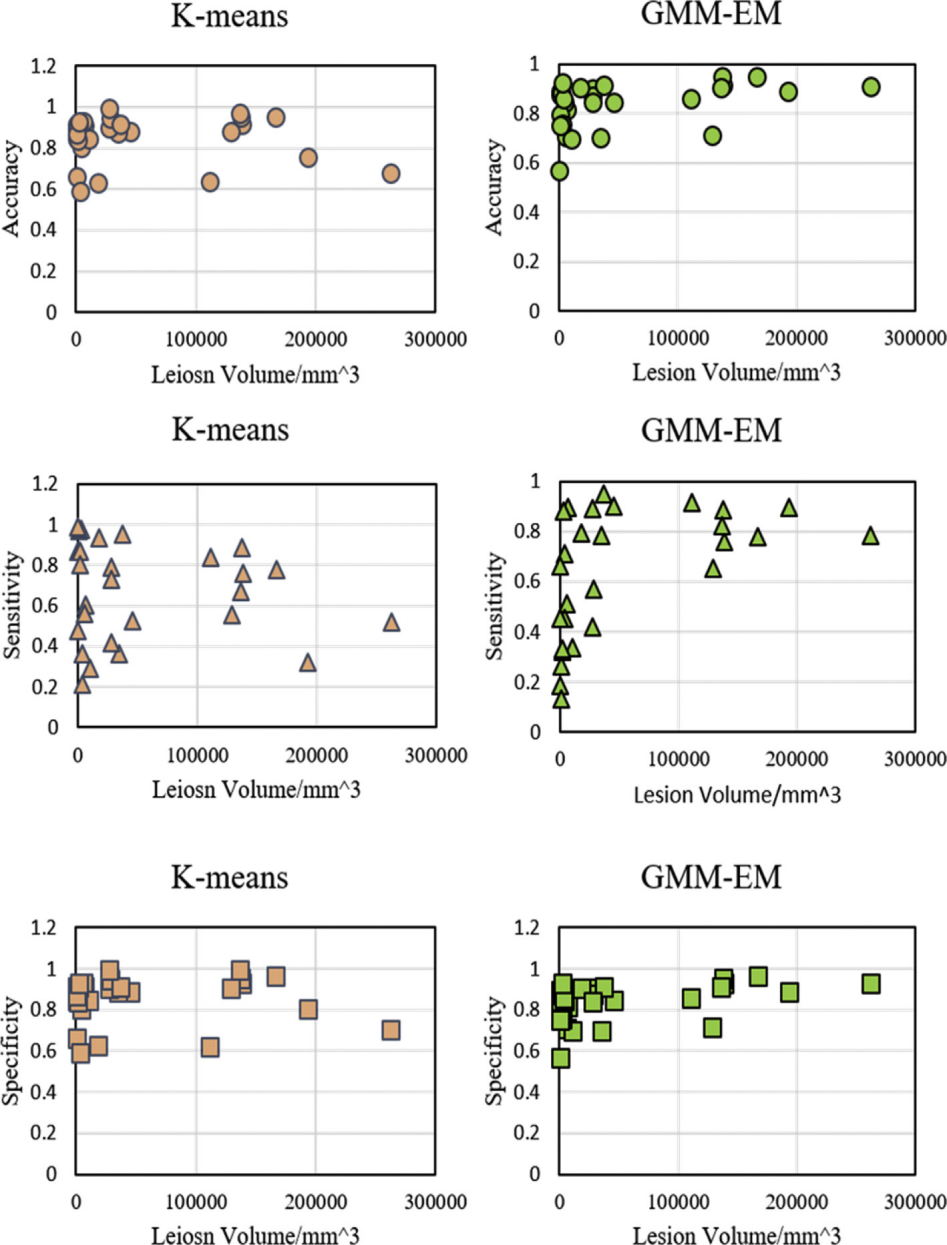
Lesion size does not affect the performance of K-means and GMM-EM algorithms.

When the algorithms were first applied to perform lesion segmentation, the intuition might suggest that the bigger the lesion size, the better the algorithms performance. However, Fig. 5 shows that there is little correlation between algorithms performance accuracy (sensitivity, and specificity) and the lesion volume. In Fig. 5, the lesion volumes were calculated by counting the number voxels labeled as lesion in mask imaging.

## 2 Experimental design, materials, and methods

### 2.1 Data and feature extraction

**Fig. 6:**
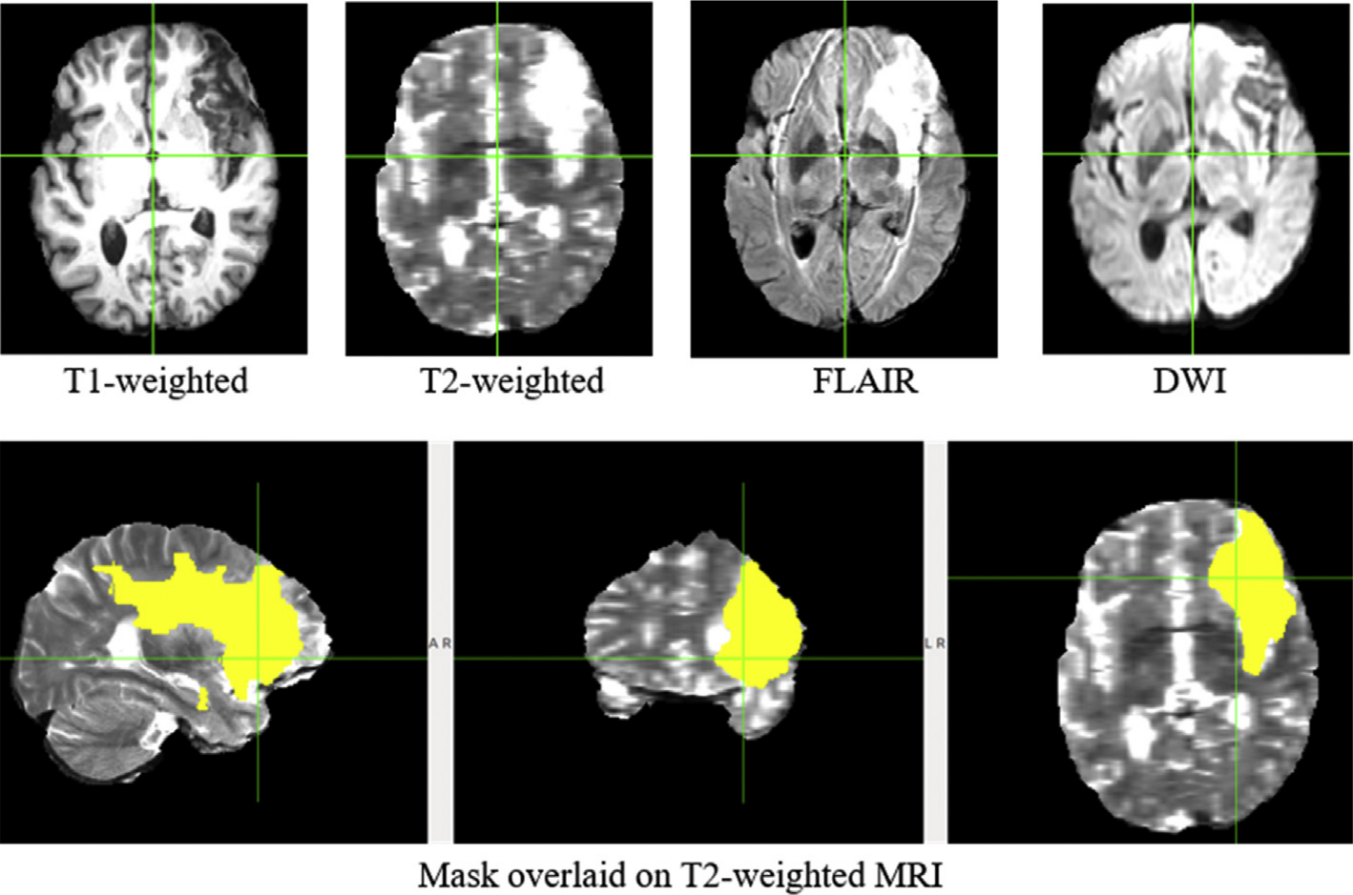
MRI data acquired from ischemic stroke lesion segmentation 2015 online database. Images from 4 MRI modalities, T1-weighted, T2-weighted, FLAIR, and DWI, are available (first row), along with mask (ground truth in yellow) overlaid on T2-weighted image in different views (second row).

Raw data were acquired from ischemic stroke lesion segmentation 2015 online database [2] (http://www.isles-challenge.org/ISLES2015/), and data is one of the two sub-taskes: sub-acute ischemic stroke lesion segmentation (SISS) training data with 28 subjects. Each of the 28 subjects contains T1-weighted, T2-weighted, FLAIR, DWI images and a lesion mask labeled by experts as ground truth as shown in Fig. 6.

**Fig. 7:**
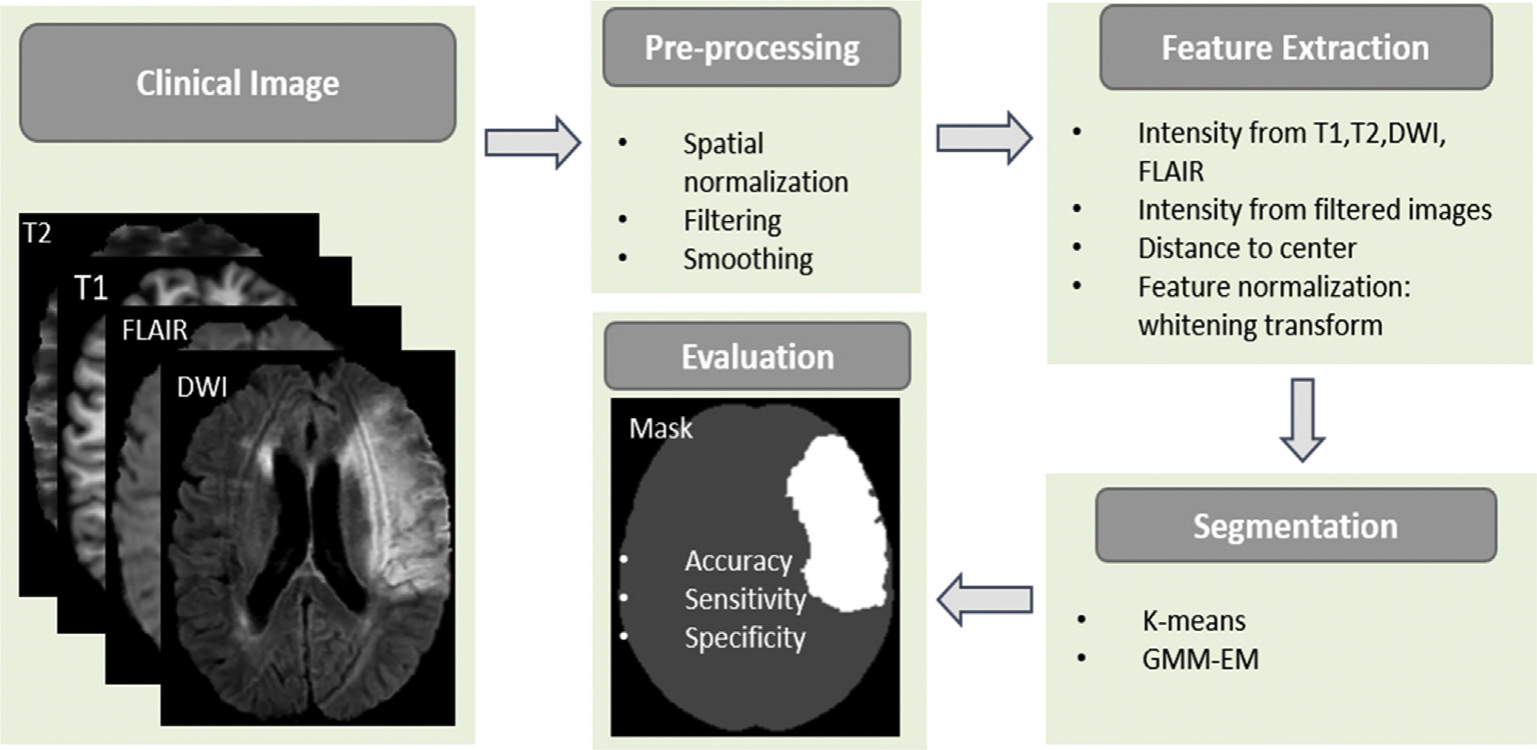
Flowchart of lesion segmentation in MRI. Four kinds of images from T1-weighted, T2-weighted, FLAIR and DWI MRI modalities were acquired and pre-processed, then features were extracted. K-means and GMM-EM algorithms were used to segment Lesion. Algorithms performance were evaluated by confusion matrix.

The flowchart of the work is shown in Fig. 7. After data were acquired, pre-processing was performed to make sure different images are in the same space. Then, features were extracted and normalized. K-means and GMM-EM were used to segment lesion from the rest of the brain tissue. Algorithms performance were evaluated by comparing the estimated lesion region with mask (ground truth).

In the pre-processing step, all images were co-registered to the standard space using MNI152 1 mm symmetric human brain atlas. In addition, for each MRI modality, images were intensity-normalized based on the average across all subjects so that features were consistent.

For each voxel, 25 features are extracted to feed into K-means and GMM-EM algorithms. The first four features are the signal intensity from T1-weighted, T2-weighted, FLAIR, DWI images. The next four are the intensities from the smoothed T1-weighted, T2-weighted, FLAIR, DWI images using a Gaussian kernel with sigma of 3 mm. Then the local information of each voxel within the brain mask is obtained using an 11 mm × 11 mm x 11mm cubic window of neighboring voxels centered at this voxel. More specifically, among more than 1 million voxels per subject, the mean, median, variance, 10th percentile and 90th percentile are calculated as four individual features for each voxel from its ±5 mm neighbors of 1330 voxels. These parameters contribute to features 9th through 24th features. The last feature was the distance of each voxel to the image center.

### 2.2 K-means clustering

K-means classifies n observations $X(x_{1},\;x_{2},\ldots ,\;x_{n)}$ into k clusters with the aim at minimizing the distance function:

$$Distance=\;\overset{k}{\underset{i=1}{\sum }}\overset{n}{\underset{j=1}{\sum }}x_{ij}-{C_{i}}^{2}$$

Where $C_{i}=\frac{1}{N_{i}}\underset{x\in x_{i}}{\sum }x,\;i=1,2,\ldots ,k$ represents the ith.

Cluster center.

The K-means algorithm:

1. Initialize cluster centroids $C_{i}$ with k random samples;
2. Assign each observation $x_{i}$ to the nearest cluster center;
3. Recalculate and update each cluster center $C_{i}=\frac{1}{N_{i}}\underset{x\in x_{i}}{\sum }x,\;i=1,2,\ldots ,k$ ; where $N_{i}$ is the number of elements in the ith cluster;
4. Repeat steps 2 and 3 until $C_{i}$ does not change.

Here, in this paper, we assign voxels into 4 groups: white matter (WM), gray matter (GM), cerebrospinal fluid (CSF), and lesion if present.

### 2.3 Gaussian mixture model-expectation maximization

In regions where MRI signal is present with signal-to-noise (SNR) ≥ 3, noise follows a Gaussian distribution approximately [[3], [4], [5]]. The histogram of brain MRI with noise in presence can be represented by a Gaussian Mixture Model in which each tissue type such as white matter, gray matter, cerebrospinal fluid, lesion if present follows a Gaussian distribution. In this model, each voxel is assigned to one of the classes.

Gaussian mixture model can be defined as:

$$p\left(x\right)=\;\overset{K}{\underset{k=1}{\sum }}\pi_{k}N\left(x|\mu_{k},\Sigma_{k}\right)$$

Where x is a d-dimensional observation vector, $\pi_{k},\;k=1,\ldots .,K\;$ are the mixture weights that satisfy $0\leq \pi_{k}\leq 1$ and $\overset{K}{\underset{k}{\sum }}\pi_{k}=1$, and $N\left(x|\mu_{k},\;\Sigma_{k}\right)$ is a d-variate Gaussian density for the kth mixture component as given by the equation:

$$N\left(x\text{|}\mu_{k},\;\Sigma_{k}\right)=\;\frac{1}{\sqrt{{\left(2\pi \right)}^{1/d}\left|\Sigma_{k}\right|}}\text{ exp}\left\{-\frac{1}{2}{\left(x-\mu_{k}\right)}^{T}{\Sigma_{k}}^{-1}\left(x-\mu_{k}\right)\right\}$$

where $\mu_{k}$ is the kth mean vector and $\Sigma_{k}$ is the kth covariance matrix.

The parameters (including means, covariances and weights of each component) can be determined by maximizing the likelihood function.

EM Algorithm.

1. Initialize means, covariances and the mixing coefficients and evaluate the initial value of the log likelihood.
2. E step. Evaluate the posterior probability using the current parameter

$$\gamma \left(z_{nk}\right)=\;\frac{\pi_{k}N(x_{n}|\mu_{k},\Sigma_{k})\;}{\sum_{j=1}^{K}\pi_{j}N(x_{n}|\mu_{k},\Sigma_{k})}$$

3. M step. Recalculate the parameters using the current posterior and update the parameters

$$\mu_{k}^{new}=\frac{1}{N_{k}}\overset{N}{\underset{n=1}{\sum }}\gamma \left(z_{nk}\right)x_{n}$$

$${\Sigma_{k}^{new}=\frac{1}{N_{k}}\overset{N}{\underset{n=1}{\sum }}\gamma \left(z_{nk}\right)\left(x_{n}-\mu_{k}^{new}\right)\left(x_{n}-\mu_{k}^{new}\right)}^{T}$$

$$\pi_{k}^{new}=\frac{N_{k}}{N}$$

where

$$N_{k}=\overset{N}{\underset{n=1}{\sum }}\gamma \left(z_{nk}\right)$$

4. Evaluate the log likelihood

$$\text{ln}p\left(X\text{|}\mu ,\Sigma ,\pi \right)=\overset{N}{\underset{n=1}{\sum }}\text{ln}\left\{\overset{K}{\underset{k=1}{\sum }}\pi_{k}N\left(x_{n}|\mu_{k},\Sigma_{k}\right)\right\}$$

5. Repeat step 2, 3, and 4 until the convergence criterion is satisfied.
